# Individually wide range of renal motion evaluated by four-dimensional computed tomography

**DOI:** 10.1186/2193-1801-3-131

**Published:** 2014-03-07

**Authors:** Hideomi Yamashita, Mami Yamashita, Masahiko Futaguchi, Ryousuke Takenaka, Shino Shibata, Kentaro Yamamoto, Akihiro Nomoto, Akira Sakumi, Satoshi Kida, Yoshihiro Kaneko, Shigeharu Takenaka, Takashi Shiraki, Keiichi Nakagawa

**Affiliations:** Department of Radiology, The University of Tokyo Hospital, 7-3-1 Hongo, Bunkyo-ku, Tokyo, 113-8655 Japan

**Keywords:** Kidney mobility, Respiration, SRT, Organ motion, Four-dimensional computed tomography

## Abstract

**Objectives:**

Assessment of physiologic renal motion in order to optimize abdominal intensity-modulated radiation therapy and stereotactic body radiation therapy.

**Methods and materials:**

Twenty patients with a median age of 47 years underwent computed tomography simulation and four-dimensional computed tomography acquisition. Thirty-nine kidneys were contoured during ten phases of respiration to estimate renal motion.

**Results:**

Kidney motion was not related to age (*p* = 0.42), sex (*p* = 0.28), height (*p* = 0.75), or body weight (*p* = 0.63). The average +/- standard deviation (SD) of movement of the center of gravity for all subjects was 11.1 +/- 4.8 mm in the cranio-caudal (CC) direction (range, 2.5-20.5 mm), 3.6 +/- 2.1 mm in the anterior-posterior (AP) direction (range, 0.6-8.0 mm), and 1.7 +/- 1.4 mm in the right-left (RL) direction (range, 0.4-5.9 mm). Renal motion strongly correlated with the respiratory phases (r > 0.97 and p < 0.01 in all three directions).

**Conclusions:**

Renal motion was independent of age, sex, height, or body weight. Renal motion in all directions was strongly respiration dependent, but motion in the cranio-caudal direction showed wide individual variation. In a clinical setting, it will be necessary to evaluate renal respiratory motion separately in each individual.

## Introduction

Inter-fractional and intra-fractional motions of critical structures are a significant concern when patients undergo intensity modulated radiotherapy. Improper dose modulation can be a result of anatomical motion. An overdosage to normal tissues can result in toxicity, whereas an under-dosage to the target tissue can lead to tumor progression. Image-guided radiotherapy has been used in an attempt to minimize the impact of anatomic motion. However, information on renal motion is scarce because of the limited amount of data available on the movement of this organ in patients undergoing radiotherapy planning. The majority of patients with renal cell carcinomas are treated during free respiration. Recently, four-dimensional computed tomography (4D-CT) simulation has enabled CT data acquisition to be gated to the respiratory cycle ((Ford et al. 2003); (Vedam et al. 2003)). This approach enables renal motion to be tracked over the entire length of the organ and in all phases within the respiratory cycle.

Because the kidney is a highly radiosensitive organ, the irradiated dose often becomes a problem when the kidney is included in the field of radiation together with the para-aortic lymph nodes, base of lung, and stomach. In addition, it is thought that the breathing-related changes of the kidney need to be considered in order to estimate the at-risk volume (PRV) of the kidney when planning stereotactic body radiotherapy (SBRT) for renal cell carcinoma (Doh et al. 2006).

## Methods and materials

### Patients

Twenty patients (15 males and 5 females) with cancer participated in this study (primary lung cancer, n = 16; metastatic lung tumor, n = 1; oropharyngeal cancer, n = 1; hepatic cell carcinoma, n = 2) (Table 1). The median age was 47 years, and ranged from 26-86 years. There was no detectable abdominal or pelvic malignancy. No patient suffered from respiratory disorders. A total of 39 kidneys were studied because one patient had a solitary kidney. This prospective study was approved by the Institutional Review Board of Tokyo University (No. 2613).

**Table 1:** **Patient characteristic**

						Surgical history					
Pt no.	Gender	Age	Hgnt (cm)	Weight (kg)	Weight/height (%)	BMI	Disease	Stage	COPD	Thoracic	Abdorminal
1	Male	39	135	115	62	33.6	Primary lung cancer	I	-	-	-
2	Male	59	167	65	39	23.2	Colon cancer	IV	+	+	+
3	Male	31	178	60	34	18.9	Primary lung cancer	I	-	-	-
4	Female	81	147	33	22	15.3	Primary lung cancer	I	-	+	+
5	Male	71	165	61	37	22.3	Primary lung cancer	I	-	-	+
6	Male	26	172	68	40	23.0	Primary lung cancer	I	-	-	-
7	Male	35	167	57	34	20.4	Primary lung cancer	I	-	-	-
8	Female	30	164	55	34	20.4	Primary lung cancer	I	-	-	-
9	Male	52	168	64	38	22.7	Primary lung cancer	I	-	-	-
10	Male	31	181	80	44	24.4	Primary lung cancer	I	-	-	-
11	Male	39	173	73	42	24.4	Primary lung cancer	I	-	-	+
12	Female	28	160	47	29	18.4	Primary lung cancer	I	-	-	-
13	Male	42	169	67	40	23.5	Primary lung cancer	I	-	-	-
14	Female	79	147	50	34	23.3	Hepatic cell carcinoma	III	-	-	+
15	Male	82	163	54	33	20.1	Orophary ngeal cancer	IV	+	-	-
16	Male	86	163	61	37	23.0	Hepatic cell carcinoma	IV	-	-	-
17	Female	77	159	48	30	19.0	Primary lung cancer	I	-	+	-
18	Male	61	175	65	37	21.2	Primary lung cancer	I	-	-	-
19	Male	26	165	53	32	19.5	Primary lung cancer	I	-	-	-
20	Male	85	150	43	28	19.0	Primary lung cancer	I	-	+	+

### 4D-CT acquisition

We obtained 4D-CT scans using a 24-detector CT simulator (Aquilion^TM^ LB, multi-slice CT system, TOSHIBA, Japan). Images were acquired in the supine position and in spiral mode by using the following parameters: 50 mA, 120 kV, 0.5-s gantry rotation, 1.5-2.0 pitch (depending on respiration rate), 1.0-mm collimation, and 2-mm slice thickness. A volume CT dose index (average absorbed dose per scan) was 93.1 mGy. Each respiratory cycle was captured as a series of eight traces, four inspiratory quarters, and four expiratory quarters acquired at equally spaced intervals between 0% and 100% during normal uncoached breathing. Breathing control methods like anesthesia, airway management, or supplemental oxygenation via nasal cannula were not used in any case.

Respiratory trace measurements were obtained with the aid of an external abdominal excursion test. An elastic belt containing a load-sensitive pressure sensor was affixed to the abdominal/low thoracic wall (Anzai Medical, Tokyo, Japan). The sensor was placed along the mid-clavicular line, approximately 5–10 cm inferior to the xiphoid process.

### Renal delineation

Bilateral renal delineation was performed for each respiratory phase (0%, 10%, 20%, …, 90%) and for each patient (total 39 kidneys of 20 patients) on a Pinnacle^3^ Ver. 9.2 treatment-planning workstation (Philips Healthcare, Andover, MA; ADAC, Milpitas, CA). The delineation was performed semi-automatically. The coordinates (x, y, and z) of the center of gravity of each kidney were calculated automatically on the workstation. We didn’t distinguish between kidney cortex and medulla in counturing. Data on the region of interest (ROI) was recorded with in-house software and an edge of 6 directions as well as the center of gravity were calculated. Positive directions were right to left on the x-axis, from posterior to anterior on the y-axis, and from superior to inferior on the z-axis. Each point of ROI was presented as the coordinate of X, Y, and Z axis. The number of ROIs in one kidney was approximate 4000 to 5000. The center of gravity was calculated as x_G_ = (m_1_x_1_ + m_2_x_2_ + … + m_n_x_n_)/(m_1_ + m_2_ + … + m_n_), y_G_ = (m_1_y_1_ + m_2_y_2_ + … + m_n_y_n_)/(m_1_ + m_2_ + … + m_n_), and z_G_ = (m_1_z_1_ + m_2_z_2_ + … + m_n_z_n_)/(m_1_ + m_2_ + … + m_n_). We regarded the data within 1 mm voxel as the same weight. The edge of 6 directions was obtained as the maximum value of all ROIs of each phase kidney.

### Statistical analyses

A linear regression model was used to investigate the relationship between dependent and independent variables. Dependent variables included measures of renal motion, whereas independent variables included age, weight, height, and diaphragmatic motion. The Fisher exact test was used to test the association between two categorical variables. Pearson correlation was used to measure the correlations among independent variables with a significance level of 0.05 for the two-sided test.

## Results

**Figure 1:**
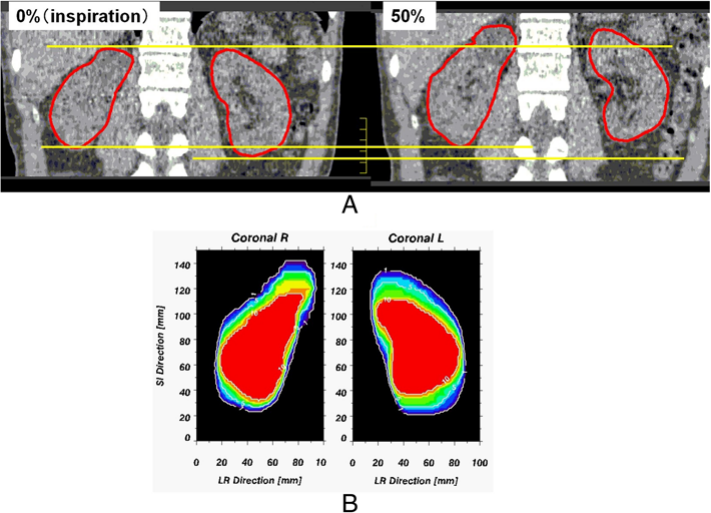
**4D-CT image taken during normal breathing in the coronal plane of patient 9.** (**A**: end-expiration and end-inspiration and **B**: renal distribution by 10 respiratory phases).

The median helical CT scan time was 68.5 seconds (range; 43.7-83.6 seconds).The average +/- SD of 39 maximum values minus minimum values for each subject on movement of the centroid was 11.1 +/- 4.8 mm in the CC direction (range, 2.5-20.5 mm), 3.6 +/- 2.1 mm in the AP direction (range, 0.6-8.0 mm), and 1.7 +/- 1.4 mm in the RL direction (range, 0.4-5.9 mm). Four-dimensional CT images of end-expiration and end-inspiration taken during normal breathing in coronal direction of Patient 9 are shown in Figure 1.

**Figure 2:**
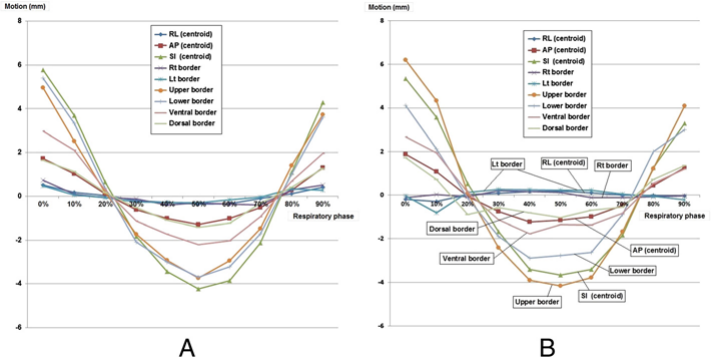
**Movements in the RL, AP, and CC directions of the centroid and of the right, left, upper, lower, ventral, and dorsal borders by respiratory phases.** (**A**: left kidney and **B**: right kidney).

The average of 19 SDs in each case on the left kidney was 0.31 mm in the RL (centroid), 1.07 mm in the AP (centroid), 3.64 mm in the CC (centroid) directions, 0.40 mm in the right border, 0.28 mm in the left border, 3.04 mm in the upper border, 3.25 mm in the lower border, 1.89 mm in the ventral border, and 1.10 mm in the dorsal border (Figure 2A and Table 2). The average on the right kidney was 0.15 mm (centroid RL), 1.11 mm (centroid AP), 3.28 mm (centroid CC), 0.13 mm (right), 0.33 mm (left), 3.82 mm (upper), 2.62 mm (lower), 1.55 mm (ventral), and 1.03 mm (dorsal) (Figure 2B and Table 2). The renal volume and motion of the centroid in the CC direction each case were independent in each case (Table 2).

**Table 2:** **Renal volume and CC motion of the centroid**

	Renal max volume (cc)		2SD of CC motion (mm)	
Pt no.	Rt	Lt	Rt	Lt
1	291	280	4.6	5.2
2	*149*	154	13.6	13.4
3	164	182	11.8	15.0
4	98	90	9.6	12.2
5	153	137	10.6	7.6
6	157	159	10.0	11.8
7	115	137	10.2	12.6
8	151	178	2.8	3.4
9	219	204	14.2	14.0
10	161	149	4.6	4.0
11	145	155	10.0	6.0
12	164	140	10.2	8.8
13	189	156	7.6	8.0
14	94	111	8.2	5.8
15	236 -	-	4.6	--
16	120	112	8.2	7.2
17	185	186	6.6	6.2
18	117	144	1.8	2.8
19	162	131	9.4	5.2
20	197	201	5.4	7.6

The movement of the kidney strongly correlated with a respiratory phase. During intake it was displaced in a caudal (*r* = 0.98 & *p* < 0.01), ventral (*r* = 0.97 & *p* < 0.01), and to an outward (*r* = 0.98 & *p* < 0.01) (Figure 2). Changes of the mean renal volume of 20 cases each phase did not correlate with respiratory phase, in other words, there was no trend that the renal volume became the minimum or maximum on inspiration or expiration.

**Figure 3:**
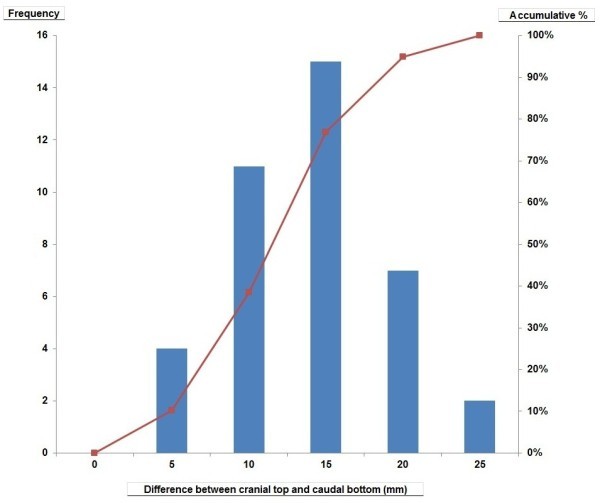
**Cumulative distribution of renal motions (=difference between cranial top and caudal bottom) in the CC direction.**

Although the factors related to the movement of the kidney were examined, none of them were significantly correlated with age (*p* = 0.42), sex (*p* = 0.28), body height (*p* = 0.75), or body weight (*p* = 0.63).The difference between cranial top and caudal bottom in the CC direction was 0-5 mm in 4 cases (accumulative rate = 10%), 5-10 mm in 11 cases (38%), 10-15 mm in 15 cases (77%), 15-20 mm in 7 cases (95%), and 20-25 mm in 2 cases (100%) (Figure 3). In the AP direction, the difference was 0-2 mm in 8 cases (21%), 2-4 mm in 17 cases (64%), 4-6 mm in 7 cases (82%), and 6-8 mm in 7 cases (100%). In the RL direction, the difference was 0-1 mm in 17 cases (44%), 1-2 mm in 11 cases (72%), 2-3 mm in 6 cases (87%), 3-4 mm in 1 case (90%), 4-5 mm in 3 cases (97%), and 5-6 mm in 1 case (100%). This movement varied among individual patients.

## Discussion

This paper is a study on kidney motion, in which 4D-CT was used as the image base and the Pinnacle workstation was used to delineate and measure the motion. By accounting for organ motion this study provides valuable information on how to avoid exposing the kidney to radiation while treating nearby targets. In addition, our data make it possible to target the kidney directly and precisely if this is the target organ. This is highly critical in SBRT for renal lesions.

The tri-axial movement of the kidney of more than 10 mm was largest in the CC direction of over 10 mm. The magnitude of this movement was larger than expected because motion in primary breast tumors was only 2.1 mm in our previous study (Yamashita et al. 2012). The head side showed the largest movement of the six edges. It had a strong association with a breathing aspect similar to that shown in previous reports ((Bussels et al. 2003); (Chen et al. 2009); (Stroom & Heijmen 2006)), and this was because the renal top end was pulled by diaphragmatic movement. After comparing the movements of right and left kidneys, there was no indication that the kidney was easily moved. The change in renal volume of the right and left kidneys had no relation to breathing.As shown in the histogram (Figure 3), the movement from smallest (2.5 mm) to largest (20.5 mm) in the CC direction of the center of gravity depicts wide individual variations. In the clinical setting, it will be necessary to evaluate renal movement with the breathing of each person at the time a radiotherapy plan is made. In this study, we did not find any determining factor (including body type or age) associated with a large renal movement.

The literature describing renal tumor motion is limited. According to Pai Panandiker et al. (Pai Panandiker et al. 2012), the 95% confidence interval for the averaged minima and maxima of renal motion in children older than 9 years revealed a wide range of motion, which was 5-16 mm in the ML direction, 6-17 mm in the AP direction, and 21-52 mm in the SI direction. Schwartz et al. (Schwartz et al. 1994) reported that the maximal vertical motion of the superior pole from its end-expiratory to its end-inspiratory position was 39 mm (43 mm for the inferior pole) and the mean deviation of kidney movement was less than 4 mm in all three dimensions (range, 0-6.9). van Sörnsen de Koste et al. (van Sörnsen de Koste et al. 2006) found that mobility was predominantly cranio-caudal, with a mean of 9.8 mm for the left kidney and 9.0 mm for the right kidney and large inter-patient variations were observed that ranged from 2.5 mm to 30 mm (left) and 2.5 mm to 20 mm (right). According to Ahmad et al. (Ahmad et al. 1997), the left kidney block required an additional 10 mm above and 15 mm below the renal silhouette on the simulation film in order to account for all phases of respiration. The corresponding values for the right kidney were 2 mm and 19 mm, respectively. In an MRI study to determine the respiration-induced motion of the kidneys, Moerland et al. (Moerland et al. 1994) found under normal respiration conditions that displacements of the left and right kidney varied from 2 mm to 24 mm and 4 mm to 35 mm, respectively. According to Siva et al. (Siva et al. 2013), the mean (interquartile range) displacement of the left and right kidneys was 0.74 cm (0.45-0.98 cm) and 0.75 cm (0.49-0.97 cm), respectively, by data sets from 71 consecutive patients with free breathing 4D-CT planning scans.

In the study by van Sörnsen de Koste et al. (van Sörnsen de Koste et al. 2006), renal motion on 4D-CT was described in 54 patients. In their study, only the cranio-caudal mobility of the upper poles of both kidneys was assessed in all 54 patients by use of the maximum translations (in the z axis) of the renal apex during all phases of the 4D-CT scans and in only 5 patients both kidneys were fully imaged on all 10 phase bins. Previous reports on renal mobility are summarized in Table 3.

**Table 3:** **Previous reports on renal mobility**

First author	Year	Ref. no.	Modality	Number	Cranio-caudal motion (mm)	
					Right	Left
Schwartz	(1994)	9	MRI	14	16 (Mean)	14 (Mean)
Moerland	(1994)	12	MRI	14	2-24	4-35
Ahmad	(1997)	11	XR	8	21	25 (Max)
Bussels	(2003)	5	MRI	12	16.1 ± 7.9	16.9 ± 6.7
Koste	(2006)	15	4D-CT	54	9.0 (2.5-20)	9.8 (2.5-30)
Brandner	(2006)	16	4D-CT	13	13 (Mean)	11 (Mean)
Panandiker	(2012)	8	4D-CT	23	12-25 (< 9 y.o)	
					21-52 (> 9 y.o)	
Our study			4D-CT	20	11.1 ± 4.8	

*Abbreviation;* MRI magnetic resonance imaging, CT computed tomography, XR X-ray.

This study has certain limiting features. With the 4D-CT method, it is difficult to examine factors that influence movement except for breathing, including the enteric peristalsis. It will be necessary to film the range of 16 cm in the cranio-caudal direction using the cine-mode of 320-row CT and to watch real renal movements in consecutive time periods. Furthermore, we studied only a small number of cases, comprising 39 kidneys of 20 cases.

Edge detection techniques in this study might be insufficient in noisy images, and it cannot distinguish between the edges of different kidney parts (e.g., cortex and medulla edges). Therefore, it is possible that more sophisticated techniques should have been used to accurately extract the kidney from CT data (Khalifa et al. 2011, 2013). Recently, a comprehensive survey on the segmentation and registration of renal data was reported by Mostapha et al. (Mostapha et al. 2013; Mostapha et al. 2014). After this first study, we will have to validate our study on a larger group of data sets in order to draw stronger conclusions. Additionally, the estimation of the kidney motion based on the center of gravity (translation) may not be accurate enough, because part of the kidney motion is a local motion, which can be accounted for by the use of more advanced motion correction techniques, such as affine registration ((Bhat et al. 2011); (Mohammadi et al. 2010)) and B-splines ((Delmon et al. 2013); (Jacobson & Murphy 2011)). Therefore, it is possible that we should have evaluated this study using more advanced registration techniques.

In a report on a series of 9 patients with primary renal cell carcinoma (RCC) treated with SBRT by Beitler et al. (Beitler et al. 2004), there were four long-term survivors (minimum follow-up of 48 months). Similar results were seen in 5 patients with primary RCC treated with SBRT and with a follow-up of more than 4 years (Wersäl1 et al. 2005). The kidney is a radio-sensitive structure. Although the kidneys move with respiration, treatment planning does not usually take into account the motion of the organs. If the renal motion is large, respiration-gated radiotherapy could be advocated in SBRT for RCC. If the movement is small, an internal target volume (ITV) that includes all movement of the gross tumor volume (GTV) by breathing could be used as irradiation target. Based on our study, we recommend evaluation of renal motion using 4D-CT and other methods in the individual patient, as well as determination of which SBRT method should be used in each case.

## Conclusion

Renal motion in the cranio-caudal direction shows wide individual variation (2.5-20.5 mm). In a clinical setting, it will be necessary to evaluate renal respiratory motion in each individual. In this study, a factor associated with renal motion was not found. Renal motion was strongly dependent on respiration, but independent of age, sex, height, and body weight. The average +/- SD of movement of the center of gravity was 11.1 +/- 4.8 mm (cranio-caudal), 3.6 +/- 2.1 mm (antero-postal), and 1.7 +/- 1.4 mm (medio-lateral).

## Abbreviations

4D: Four dimensions
CT: Computed tomography
RPV: Planning at risk volume
SBRT: Stereotactic body radiotherapy
ROI: Region of interest
CC: Cranio-caudal
AP: Anterior-posterior
RL: Right-left
SD: Standard deviation
RCC: Renal cell carcinoma
ITV: Internal target volume
GTV: Gross tumor volume.
